# Histone Acetylation as a Regenerative Target in the Dentine-Pulp Complex

**DOI:** 10.3389/fgene.2020.00001

**Published:** 2020-02-06

**Authors:** Yukako Yamauchi, Paul Roy Cooper, Emi Shimizu, Yoshifumi Kobayashi, Anthony J. Smith, Henry Fergus Duncan

**Affiliations:** ^1^ Division of Restorative Dentistry & Periodontology, Dublin Dental University Hospital, Trinity College Dublin, University of Dublin, Dublin, Ireland; ^2^ Sir John Walsh Research Institute, Faculty of Dentistry, University of Otago, Dunedin, New Zealand; ^3^ Oral Biology Department, Rutgers School of Dental Medicine, Newark, NJ, United States; ^4^ Oral Biology, School of Dentistry, College of Medical and Dental Sciences, University of Birmingham, Birmingham, United Kingdom

**Keywords:** histone deacetylases, dentinogenesis, regenerative endodontics, dental pulp, acetylation, histone acetyltransferases

## Abstract

If dental caries (or tooth decay) progresses without intervention, the infection will advance through the dentine leading to severe pulpal inflammation (irreversible pulpitis) and pulp death. The current management of irreversible pulpits is generally root-canal-treatment (RCT), a destructive, expensive, and often unnecessary procedure, as removal of the injurious stimulus alone creates an environment in which pulp regeneration may be possible. Current dental-restorative-materials stimulate repair non-specifically and have practical limitations; as a result, opportunities exist for the development of novel therapeutic strategies to regenerate the damaged dentine-pulp complex. Recently, epigenetic modification of DNA-associated histone ‘tails’ has been demonstrated to regulate the self-renewal and differentiation potential of dental-stem-cell (DSC) populations central to regenerative endodontic treatments. As a result, the activities of histone deacetylases (HDAC) are being recognised as important regulators of mineralisation in both tooth development and dental-pulp-repair processes, with HDAC-inhibition (HDACi) promoting pulp cell mineralisation *in vitro* and *in vivo*. Low concentration HDACi-application can promote de-differentiation of DSC populations and conversely, increase differentiation and accelerate mineralisation in DSC populations. Therapeutically, various HDACi solutions can release bioactive dentine-matrix-components (DMCs) from the tooth’s extracellular matrix; solubilised DMCs are rich in growth factors and can stimulate regenerative processes such as angiogenesis, neurogenesis, and mineralisation. The aim of this mini-review is to discuss the role of histone-acetylation in the regulation of DSC populations, while highlighting the importance of HDAC in tooth development and dental pulp regenerative-mineralisation processes, before considering the potential therapeutic application of HDACi in targeted biomaterials to the damaged pulp to stimulate regeneration.

## Introduction

Dental caries (decay) is the most prevalent global non-communicable disease (WHO, 2017). The caries process initiates with a microbial biofilm forming on the tooth surface, which ‘fuelled’ by a dietary source of fermentable carbohydrates, ecologically shifts the plaque to an acidogenic flora, breaking down the hard tooth tissues of enamel and dentine (Nyvad et al., 2013). If the carious lesion progresses without remedial treatment, the pulp tissue in the centre of the tooth will become progressively infected and inflamed (Mjor and Tronstad, 1972; Mjor and Tronstad, 1974). The pulpal inflammation (pulpitis) provokes a robust defensive reaction with new dentine produced by the pulp’s secretory cells, the odontoblasts, locally beneath the caries in a process called reactionary dentinogenesis (Smith, 2002). If the advancing caries continues until the bacteria invade the pulp tissue, odontoblast death will occur, prior to more widespread pulpal necrosis. Traditional treatment for pulp necrosis is root-canal-treatment (RCT) (**Table 1**), which effectively removes all pulp tissue; however, this is a very destructive and empirical approach. The absence of vital pulp tissue has other consequences, including removal of the tooth’s developmental, reparative, and immune capacity as well as loss of the pulps proprioceptive sensors, accompanied by a significantly greater risk of fracture and tooth loss (Paphangkorakit and Osborn, 1998; Smith, 2002). The pulp; however, has considerable potential to regenerate if the insult is removed and the tooth effectively restored during vital-pulp-treatment (VPT) (Mjor and Tronstad, 1974). The damaged odontoblast layer can regenerate in a stem-cell (SC) led process, in which stem/progenitor cells cyto-differentiate under the influence of bioactive molecules released from the damaged dentine and pulp cells (Lesot et al., 1994; Smith et al., 2016; Neves et al., 2017). Unfortunately, current therapies, which aim to maintain and regenerate the pulp in VPT, are limited by low-quality hard-tissue formation and non-specific responses (Nair et al., 2008; Sangwan et al., 2013). As a result, there is significant interest in developing scientific understanding of the mechanisms that control dental SC (DSC) fate as well as identifying potential therapeutic targets to promote more effective tissue regenerative processes.

**Table 1:** A list of abbreviations and definitions used in the text and figures.

Abbreviations	Definition
BDNF	Brain-derived neurotrophic factor
BMP	Bone morphogenetic protein
DFPC	Dental follicle progenitor cell
DMC	Dentine matrix component
DMP	Dentin matrix acidic phosphoprotein 1
DPC	Dental pulp cell
DPSC	Dental pulp stem cell
DSC	Dental stem cell
DSPP	Dentin sialophosphoprotein
ESC	Embryonic stem cell
FDA	US Food and Drug Administration
GDF-15	Growth/differentiation factor 15
GF	Growth factor
GNAT	GCN5-related *N*-acetyltransferases
HAT	Histone acetyl transferase
HDAC	Histone deacetylase
HDACi	Histone deacetylase inhibitor
LMK-235	N-[[6-(hydroxyamino)-6-oxohexyl]oxy]-3,5-dimethyl-benzamide
MMP	Matrix metalloproteinase
MYST	MOZ, YBF2/SAS3, SAS2, and TIP60
PDLC	Periodontal ligament cell
RCT	Root canal treatment
SAHA	Suberoylanilide hydroxamic acid
SC	Stem cell
TGF	Transforming growth factor
TSA	Trichostatin A
VPA	Valproic acid
VPT	Vital pulp treatment

Epigenetic modulations, DNA-methylation and histone modifications, are important regulators of DSC fate (Gopinathan et al., 2013), with histone acetylation being identified as an important regulator of bone, periodontal ligament, and dental pulp mineralisation processes as well as being a target for therapeutic inhibition (Duncan et al., 2016; Huynh et al., 2016; Ricarte et al., 2016; Cantley et al., 2017). The acetylation of DNA-associated histone (and non-histone) proteins is controlled by the enzymes histone-deacetylases (HDACs) and histone-acetyl-transferases (HATs), which alter chromatin architecture in response to cellular needs, regulating transcription (Kouzarides, 2007). HATs or lysine acetyltransferases, are bi-substrate enzymes, which are generally divided into categories of which the GCN5-Related *N*-Acetyltransferases (GNAT) and MYST families the largest, although others such as CBP/p300 may also be functionally important (Lalonde et al., 2014). HATs are further classified by their nuclear or cytoplasmic distribution (Richman et al., 1988) and have been implicated in a range of inflammatory diseases (e.g. asthma) and cancer (Ito et al., 2002; Yang, 2004). To date, HATs have not been the focus of the same level of attention as HDACs in regenerative medical or dental research and although HAT inhibitors are available, *in vitro* performance has not been replicated therapeutically (Wu et al., 2009; Lasko et al., 2017). This has been attributed to the difficulty in designing effective HAT inhibitors, as they influence a range other cellular substrates and operate as part of multi-function complexes (Wapenaar and Dekker, 2016).

There are eighteen human HDAC enzymes categorised into four separate classes, with classes I, II, and IV containing zinc-dependent enzymes (Seto and Yoshida, 2014). Class I HDACs demonstrate ubiquitous expression, while class II show tissue-specific expression and cellular localisations (Montgomery et al., 2007). The importance of class II HDAC expression in mineralising tissues has been demonstrated in bone (Ricarte et al., 2016) and teeth (Klinz et al., 2012), with the individual isoforms, -6 (Westendorf et al., 2002), -5, and -4 (Nakatani et al., 2018), highlighted as being important cellular mediators which regulate osteoblast differentiation. HDACs’ roles in the regulation of mineralisation and developmental cellular processes (Gordon et al., 2015), also make them attractive therapeutic targets for pharmacological inhibition (Richon et al., 1996). Several HDAC inhibitors (HDACis), including trichostatin A (TSA), valproic acid (VPA), and suberoylanilide hydroxamic acid (SAHA), have been shown to have clinical application in a range of diseases including cancer and inflammatory and neurodegenerative disorders (Bolden et al., 2006; Das Gupta et al., 2016; Naftelberg et al., 2017). The medical and dental literature also reports that HDACis are associated with anti-inflammatory effects, pro-mineralisation, increased SC differentiation, and overall improved regenerative responses (Halili et al., 2009; Xu et al., 2009; Wang et al., 2010; Duncan et al., 2013; Luo et al., 2018). Consequently, HDACis have the potential to enhance dentine regenerative processes in VPT by directly influencing DSC populations (Duncan et al., 2012; Luo et al., 2018) and indirectly, by inducing the solubilisation of dentine matrix components (DMCs) rich in growth factors (GFs) and other bioactive molecules (Smith et al., 2016; Duncan et al., 2017). An emerging role for HDACs in tooth development and regeneration presents an opportunity for HDACi use in novel dental regenerative materials.

The following section of this mini-review is to discuss specifically the role of histone-acetylation in the regulation of DSC populations, while highlighting the importance of HDAC in tooth development (primary dentinogenesis) and dental pulp regenerative-mineralisation processes (tertiary dentinogenesis). Finally, the therapeutic regenerative potential of a topically applied HDACi as part of next-generation dental biomaterials to regenerate the damaged pulp is considered.

## Review

### The Need to Regenerate Dental Pulp Tissue

The tooth consists of the outermost enamel and inner dentine, which surround a centrally-placed connective tissue called the pulp. Enamel is a highly mineralised tissue produced by the ameloblast cell during tooth development; however, after eruption, enamel has no cellular capacity to continue development, repair, or regenerate. Dentine is formed by the secretory odontoblast cells, which reside at the interface between dentine and pulp, linking the two tissues in a structure that is known as the dentine-pulp-complex (Pashley, 1996). Primary dentine forms during tooth development; however, unlike enamel, secondary dentine continues to form throughout the life of the tooth and furthermore the tooth can repair damaged tissue by forming tertiary dentine in response to injurious stimuli, including caries or tooth wear (Lesot et al., 1994; Smith, 2002). There are two types of tertiary dentine, with reactionary dentine formed in response to mild to moderate irritation due to the upregulation of existing primary odontoblast activity and reparative dentine generated when severe irritation leads to odontoblast death followed by the regeneration of a new layer of odontoblast-like cells from SCs (Lesot et al., 1994).

The origin of the progenitor cells in reparative dentinogenesis is mesenchymal (Simon and Smith, 2014). Attributed to SC populations within the pulp (e.g. dental-pulp-SCs [DPSCs]) (Smith and Lesot, 2001), SCs migrating from outside the tooth (Feng et al., 2011; Frozoni et al., 2012) or undifferentiated mesenchymal cells from cell-rich and central pulp perivascular regions (e.g. pericytes) (Fitzgerald et al., 1990; Machado et al., 2016). DPSCs, reportedly account for between 1 and 5% of total permanent pulpal cells (Gronthos et al., 2000) and reside in perivascular areas potentially enabling their mobilisation to wound sites (Shi and Gronthos, 2003; Crisan et al., 2008; Casagrande et al., 2011). The dentine stores a plethora of bioactive DMCs including GFs, chemokines, bioactive-proteins, tissue proteases, and other mobilisation factors, which are released by the caries process and orchestrate healing contributing to regenerative process in the tooth (Smith, 2003; Smith et al., 2016; Duncan et al., 2017; Tomson et al., 2017). Certain dental materials exhibit the ability to solubilise DMCs and influence the quality of the new mineral tissue formed, with the outcome of VPT dependent on the dental biomaterial placed in contact with the pulp (Nair et al., 2008). Notably, calcium-silicate materials, such as mineral-trioxide-aggregate have now superseded calcium hydroxide (Bjorndal et al., 2017) as the VPT material of choice (Hilton et al., 2013). However, all current materials are limited by low-quality tertiary dentine formation, non-specific actions, and the absence of targeted components focused on tissue regenerative strategies (Duncan et al., 2011).

Regeneration processes within the dentine-pulp-complex require the presence of vital pulp tissue; however, if the inflammatory process is allowed to continue without treatment, pulp necrosis results. Regenerative endodontic efforts to avoid RCT and ‘regrow’ the dental pulp using either a SC-based (Iohara et al., 2011) or cell-homing (Shimizu et al., 2012) technique have demonstrated that pulpal regeneration is possible. DPSCs can be transplanted *in vivo* with a scaffold to form a new physiologically functioning pulp tissue (Nakashima et al., 2017) and although, development is hampered by expense, risk of immune-rejection, ethics, and other regulatory issues (Kim et al., 2013) these therapies have proceeded to clinical trial stage (Nakashima and Iohara, 2017). In an alternative revitalisation procedure, a decellularised or synthetic scaffold containing bioactive molecules such as GFs, pharmacological inhibitors, and mobilisation factors is placed into the root canal and endogenous SCs are ‘homed’ into the space before undergoing differentiation (Galler, 2016). Although, revitalisation can successfully develop a biological pulp replacement, current protocols do not specifically regenerate the odontoblast layer or indeed enable further tooth root growth, which may be necessary in under developed teeth (Shimizu et al., 2013; Eramo et al., 2018).

There is significant need to develop regenerative endodontic techniques by developing our understanding of the epigenetic processes, which control the fate and the odontogenic potential of various SC populations (Gopinathan et al., 2013; Ching et al., 2017). Histone acetylation is an obvious focus, playing a critical role in a wide range of biological processes including inflammation, mineralised tissue formation, and SC regulation, (Schroeder and Westendorf, 2005; Shuttleworth et al., 2010; Jamaladdin et al., 2014) and can be targeted by HDACi, potentially benefiting the regenerative response within VPT (de Boer et al., 2006; Duncan et al., 2016).

### Histone Acetylation Regulation of Regenerative Mineralisation Processes

The nucleosome consists of tightly-coiled DNA, wrapped around a histone core. The core contains an octamer of histone proteins (H2A, H2B, H3, and H4), each with a positively charged N-terminal tail (Biswas et al., 2011). These tails extend from the core structure, facilitating post-translational modification by acetylation, methylation, phosphorylation, ubiquitination, and SUMOylation (Zhang and Reinberg, 2001). Histone acetylation generates an architecturally open chromatin structure, which is transcriptionally active, while deacetylation tightens the DNA-histone association and represses gene expression (Verdone et al., 2005). The enzymes, HAT and HDAC, mediate these processes. Histone modifications, in contrast to DNA-methylation, are highly labile, presenting attractive targets for therapeutic intervention (Kelly et al., 2010).

**Figure 1:**
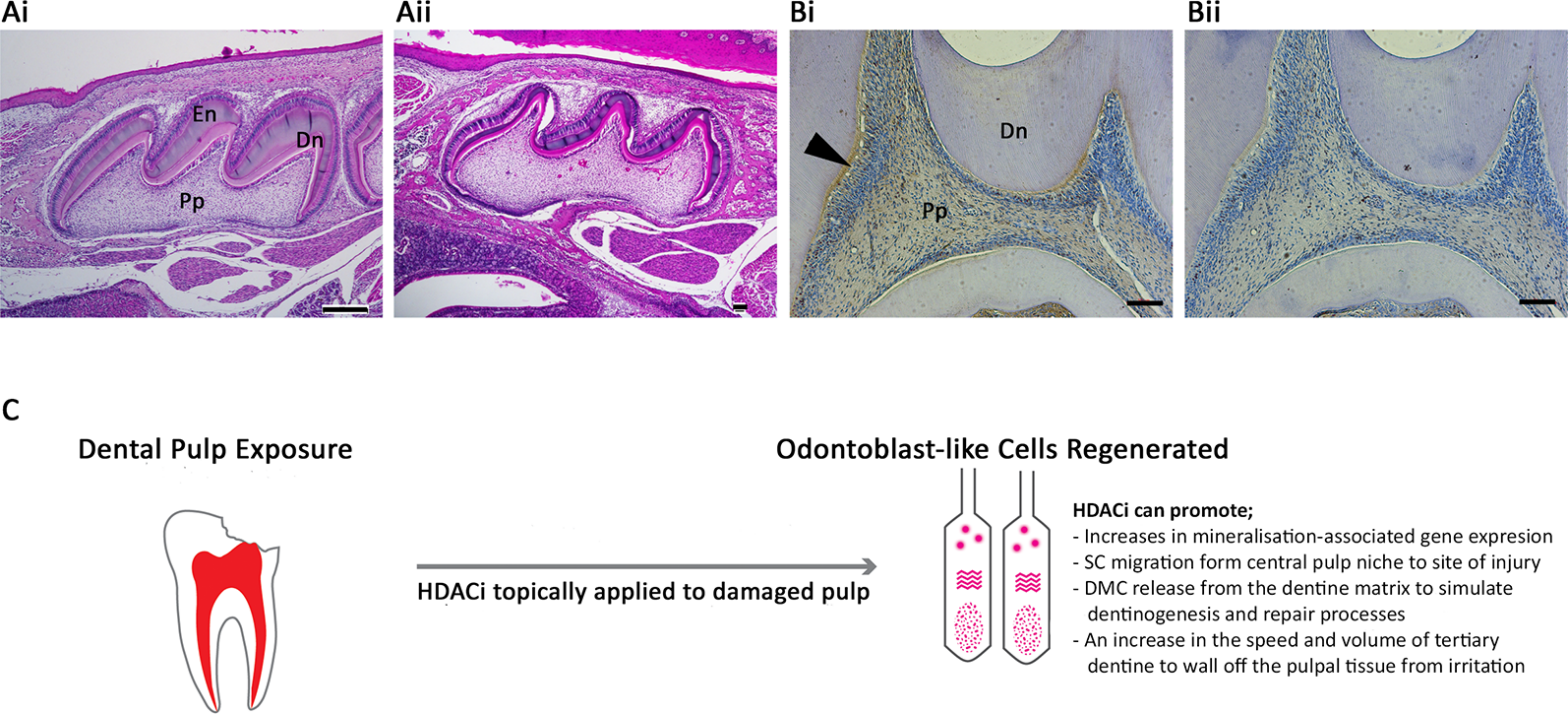
Histone acetylation as a potential therapeutic target within the dentine-pulp complex. **(A)** Morphological comparison of post-natal day 10 maxillary first molar teeth of (Ai) WT and **(Aii)** HDAC4−/− mice using haematoxylin and eosin staining of sagittal sections highlighting differences in the volume of dentine and enamel deposited in the crown of the tooth. **(Bi)** Immunohistochemical analysis demonstrating HDAC-5 expression was evident in the odontoblasts (arrow), predentine layer, and pulp of WT adult first molar teeth in rats compared with **(Bii)** negative control. Dn = mineralised dentine; En = enamel; Pp = pulp tissue. Scale bars = **(Ai)** 250 μm, **(Aii)** 10 μm (original magnification x4), **(Bi-ii)** 50 μm (original magnification x10) (Duncan, 2017) **(C)** Schematic illustration of the potential of HDACi to be applied topically to damaged pulp tissue in a dental procedure to promote regenerative responses in VPT. Odontoblast-like cells are a replacement secretory cell after the death of primary odontoblast cells, which have been lost during the traumatic or carious insult. The differentiation of this cell type is crucial to the regeneration of dentine and mineralised tissue within the dentine-pulp complex. HDACi have been shown to augment several cellular processes central to this regenerative process, including increasing odontogenic gene expression, stimulating stem cell migration, promoting the release of bioactive dentine matrix components and accelerating mineralisation. SC, stem cell; DMC, dentine matrix component.

Notably altered HDAC expression occurs during osteogenesis (Westendorf et al., 2002; Schroeder et al., 2004), dentinogenesis (Klinz et al., 2012), and cementogenesis (Huynh et al., 2016) in a tissue-specific manner. Class I and II HDAC expression analysed in human tooth periodontal ligament cell (PDLC) cultures demonstrated that all of the five HDACs studied (HDAC-1 to -4 and -6) were highly expressed, although HDAC3 was downregulated during osteogenic differentiation (Huynh et al., 2016). Furthermore, a dental pulp study analysing extracted adult human molar teeth demonstrated that HDAC-2 and -9 were expressed in DPC, and exhibited a relatively strong expression in odontoblasts, while HDAC-1, -3, and -4 were relatively weakly expressed within the pulp tissue (Klinz et al., 2012). In the developing tooth, the role of histone methylation and demethylation has been studied (Zheng et al., 2014; Yi et al., 2016); however, currently little is known about the influence of acetylation in this process. Several studies have investigated the importance of HDACs in pulpal mineralisation processes and odontoblast differentiation *in vitro* (Duncan et al., 2012; Duncan et al., 2013; Paino et al., 2014), but further work is required to understand HDACs role during tooth development *in vivo*. Deletion of HDAC-4 in mice inhibited bone resorption and reduced thickness and cortical bone mass (Nakatani et al., 2016), and had the additional effect of inhibiting MMP-13 and *Sost/sclerostin* expression (Nakatani et al., 2018). Dentally, a mouse model of HDAC-4 KO demonstrated altered mineralisation in the roots of developing teeth (Ono et al., 2016) and the volume of enamel and dentine (**Figure 1A**). Other histological work has highlighted strong expression of another class II HDAC, -5, in the odontoblasts of developing teeth (**Figure 1B**). Supplementing DPSC cultures with HDACi has also indicated the importance of HDAC-3 downregulation during odontoblast differentiation (Jin et al., 2013), while HDAC-2 silencing in DPSCs promoted matrix mineralisation and related gene expression (Paino et al., 2014).

HDAC and HAT activity preserves the self-renewal capabilities of mesenchymal SCs (Romagnani et al., 2007; Lee et al., 2009; Jamaladdin et al., 2014) by maintaining expression of key pluripotent transcription factors, which are required to enable an open chromatin structure characteristic of embryonic SC (ESC) populations (Jamaladdin et al., 2014). Dental pulp tissue in adult teeth contains a characterised post-natal SC population of DPSCs (Gronthos et al., 2000) and as a result, modulators of SC behaviour have attracted significant interest in dentistry with suggestions that dental developmental anomalies, including dentine dysplasia and dentinogenesis imperfecta, may be related to dysregulated epigenetic modifications present during odontoblast differentiation (Sun et al., 2015). Epigenetic modifications and related differentiation profiles of two dental SC populations, DPSCs and dental follicle progenitor cells (DFPCs), were compared *via* the analysis of odontogenic gene expression including dentine sialophosphoprotein (DSPP) and dentin matrix acidic phosphoprotein 1 (DMP-1) (Gopinathan et al., 2013). Transcript levels were epigenetically-suppressed in DFPCs, while osteogenic stimulation *in vitro* demonstrated significant mineralisation increases only in DPSCs (Gopinathan et al., 2013). Notably, a highly dynamic histone modification response was demonstrated in mineralising DFPCs, but not in DPSCs, with the latter also expressing relatively high levels of the pluripotency-associated transcripts, *Oct4* and *Nanog*. It was concluded that these two neural crest-derived SC populations were distinguished by epigenetic repression of dentinogenic genes with dynamic histone enrichment in DFPCs during mineralisation. This study highlighted the potential important role of epigenetic control in odontoblasts.

HDAC role in modulation of immune and inflammatory responses are also emerging (Leoni et al., 2002; Shanmugam and Sethi, 2013; Das Gupta et al., 2016), as well as, their role in angiogenesis (Mahpatra et al., 2010; Tsou et al., 2016) and neurogenesis (Cho and Cavalli, 2014), which are critical to the promotion of regenerative processes in the dental pulp. Together, these studies highlight that HDACs are involved in range of cellular events associated with the regeneration of dentine-pulp complex, suggesting their potential roles as therapeutic targets for VPT.

### HDACi in Regenerative Endodontic Therapies

HDACis chemically include short-chain fatty acids, hydroxamic acids, cyclic peptides, and benzamides (Dokmanovic et al., 2007; Marks and Xu, 2009; Yusoff et al., 2019). VPA is a short-chain fatty acid that weakly inhibits class I and IIa HDACs, while the common hydroxamic-acid-based HDACis target classes I and II HDACs. HDACi are prime discovery targets for introduction into clinical trials including SAHA (Richon et al., 1998), also known as Vorinostat, being the first HDACi to obtain US FDA-approval in 2006 for treatment of lymphoma (Grant et al., 2007). Although HDACs are critical to the control of transcription, less than 5% of expressed genes are altered by low-dose HDACi in primary DPC cultures (Duncan et al., 2016).

Pharmacological inhibition of HDACs can modulate dental-derived SC populations and promote odontoblast-like cell differentiation and mineralised tissue formation (Kwon et al., 2012; Duncan et al., 2013; Paino et al., 2014). Application of pan-HDACi, TSA, VPA, and SAHA, to rodent and human DPSC cultures enhanced mineralisation, accompanied by an upregulation of genes associated with odontoblast differentiation and mineralisation, including *TGF-β1*, bone morphogenic proteins (BMPs), *DMP*, and *DSPP* (Duncan et al., 2012; Duncan et al., 2013; Paino et al., 2014; Duncan et al., 2016). In contrast to the general upregulation of mineralisation-associated transcripts, the expression of the bone metabolism marker osteocalcin was reduced, a result attributed to the use of VPA (Jin et al., 2013; Paino et al., 2014). HDACis reduced cell proliferation and viability at relatively high doses, but at lower doses did not show cytotoxic or anti-proliferative effects (Duncan et al., 2013; Paino et al., 2014; Duncan et al., 2017). SAHA was also shown to promote other reparative processes in DPC populations, including cell migration (Duncan et al., 2016; Luo et al., 2018) and cell adhesion (Luo et al., 2018). In addition to the direct regulation of SCs, HDACis also induce bioactive DMC release from dentine (Duncan et al., 2017). Bioactive molecules ‘fossilised’ within the dentine matrix (Cassidy et al., 1997; Smith, 2003; Grando Mattuella et al., 2007), can be released by caries, trauma, or by dental materials (Graham et al., 2006; Tomson et al., 2007). Released DMCs regulate the cyto-differentiation of progenitor cells and subsequent reparative dentine formation with bioactive components including BMPs and other GFs (Smith et al., 2016). Three HDACis, SAHA, TSA, and VPA, extracted a range of GFs from dentine, less efficiently than the well-characterised extractant EDTA for certain GFs (e.g. TGF-β1), but more effectively for others (e.g. Growth/differentiation factor 15 [GDF-15], Brain-derived neurotrophic-factor [BDNF]), while interestingly each HDACi exhibited a different extraction profile (Duncan et al., 2017). Furthermore, an *in vivo* study analysed the development of the dentine-pulp complex after systemic injection of TSA into prenatal mice and highlighted an increase in odontoblast numbers and dentine thickness compared with control specimens (Jin et al., 2013).

Currently, most research in medicine and dentistry employs pan-inhibitors; however, isoform-specific HDACis have been developed (Khan et al., 2008; Muraglia et al., 2008). It is proposed that isoform selectivity will counteract the multiple, often opposing cellular effects of HDACs (Balasubramanian et al., 2009) and reflect tissue-dependent expression of class II HDAC enzymes in particular (Verdin et al., 2003). For example, LMK-235 selectively inhibits HDAC-4 and -5 and was reported to upregulate odontoblast differentiation from human DPSCs (Liu et al., 2018). From a therapeutic perspective, low-dose short-duration HDACi application promotes DPC regenerative processes highlighting an opportunity for its use in next-generation VPT biomaterials (Duncan et al., 2016). Ethical, regulatory, and cost-effectiveness appraisal will need to be considered and material science aspects developed in order to create a controlled delivery-mechanism for the pulp. Notably, dental biomaterials containing antibiotics are commercially available (Imazato et al., 2007; Kamocki et al., 2015). Certainly, the low-dose, topical route of administration in dentistry should reduce the likelihood of systemic side effects such as fatigue, nausea, vomiting, diarrhoea, and thrombocytopenia (Subramanian et al., 2010), which have been reported following systemic-administration of HDACis at high-dose and frequency for cancer therapy.

## Conclusion

A range of HDACs are expressed in the dentine-pulp complex and pharmacologically targeting them promotes a range of regenerative processes in DPC populations. Acetylation is central to orchestrating the differentiation and de-differentiation potential of DPSCs and understanding the intricacies of this control is crucial to enable pulpal regenerative responses as well as for designing novel therapeutic solutions. Further translational research is required to address clinical application and safety concerns in combination with scientific research to understand the mechanisms of epigenetic regulation of DPSC populations.

## Author Contributions

YY searched the literature, wrote and edited the manuscript. PC and AS provided guidance and edited the manuscript. ES and YK provided guidance, contributed to the figures and edited the manuscript. HD planned, provided guidance, wrote sections, contributed to the figures and edited the manuscript.

## Conflict of Interest

The authors declare that the research was conducted in the absence of any commercial or financial relationships that could be construed as a potential conflict of interest.
